# Association between ovarian reserve and utilization of preimplantation genetic testing for aneuploidy in autologous IVF cycles

**DOI:** 10.1093/hropen/hoag052

**Published:** 2026-06-02

**Authors:** David H Barad, Sarah Darmon, Sonia Gayete-Lafuente, Lara Guijarro-Baude, David F Albertini, Pasquale Patrizio, Cari Nicholas, Norbert Gleicher

**Affiliations:** The Center for Human Reproduction (CHR), New York, NY, USA; The Foundation for Reproductive Medicine, New York, NY, USA; The Center for Human Reproduction (CHR), New York, NY, USA; The Center for Human Reproduction (CHR), New York, NY, USA; The Foundation for Reproductive Medicine, New York, NY, USA; The Center for Human Reproduction (CHR), New York, NY, USA; Department of Obstetrics and Gynecology, Hospital Universitari de Vic, Barcelona, Spain; The Center for Human Reproduction (CHR), New York, NY, USA; Department of Reproductive Cell Biology, Bedford Research Foundation, Bedford, MA, USA; The Center for Human Reproduction (CHR), New York, NY, USA; Department of Obstetrics, Gynecology and Reproductive Sciences, University of Miami, Miller School of Medicine, Miami, FL, USA; The Center for Human Reproduction (CHR), New York, NY, USA; The Center for Human Reproduction (CHR), New York, NY, USA; The Foundation for Reproductive Medicine, New York, NY, USA; Stem Cell Biology and Molecular Embryology Laboratory, Rockefeller University, New York, NY, USA; Department of Obstetrics and Gynecology, Vienna University School of Medicine, Vienna, Austria

**Keywords:** anti-Müllerian hormone, SART CORS, ovarian reserve, preimplantation genetic testing for aneuploidy, IVF, ART, embryo selection, selection bias, reproductive aging, autologous IVF

## Abstract

**STUDY QUESTION:**

Does ovarian reserve, as measured by anti-Müllerian hormone (AMH), influence the utilization of preimplantation genetic testing for aneuploidy (PGT-A) in autologous IVF cycles across different age groups?

**SUMMARY ANSWER:**

Lower ovarian reserve, reflected by reduced AMH levels, was associated with a lower likelihood of undergoing PGT-A across all age strata.

**WHAT IS KNOWN ALREADY:**

PGT-A is widely used in IVF, particularly in older patients, but its utilization may be influenced not only by age but also by expected ovarian reserve and embryo yield. How ovarian reserve is associated with selection for PGT-A at a population level has not yet been well characterized.

**STUDY DESIGN, SIZE, DURATION:**

This cross-sectional analysis of the US Society for Assisted Reproductive Technology Clinic Outcome Reporting System (SART CORS) database includes 258,532 patient-first autologous ovarian stimulation cycles performed between 2014 and 2021.

**PARTICIPANTS/MATERIALS, SETTING, METHODS:**

The study included the first autologous IVF cycles of women aged 21–46 years. Donor oocyte, donor embryo, and gestational carrier cycles were excluded, as were cycles using <150 IU/day of gonadotropins or with missing key data. Cycles were classified according to PGT-A use. Ovarian reserve was primarily assessed using the most recent AMH value measured within 1 year of treatment. Multivariable logistic regression models evaluated the association between AMH and PGT-A utilization, adjusting for age, calendar year, race, total gonadotropin dose, gravidity, and prior ART, with prespecified testing for the age–AMH interaction.

**MAIN RESULTS AND THE ROLE OF CHANCE:**

Across the study population, higher AMH levels were associated with significantly increased odds of undergoing PGT-A. This association persisted after multivariable adjustment and within each age group. AMH modelled as both a continuous variable and dichotomized at 1 ng/ml showed consistent results, indicating an independent relationship between ovarian reserve and PGT-A utilization that was unlikely due to chance.

**LIMITATIONS, REASONS FOR CAUTION:**

The observational, cross-sectional design limits causal inference. Residual confounding is possible, and registry data do not capture treatment intent or all of the clinical reasons underlying use or non-use of PGT-A. Embryo-level genetic outcomes and clinical outcomes such as live birth were not evaluated.

**WIDER IMPLICATIONS OF THE FINDINGS:**

These findings suggest a systematic selection pattern in IVF practice, whereby patients with diminished ovarian reserve are less likely to undergo PGT-A. This population imbalance should be considered when interpreting observational studies of PGT-A and when counselling patients about the clinical context in which PGT-A is most commonly used.

**STUDY FUNDING/COMPETING INTEREST(S):**

This work was supported by intramural funds from The Center for Human Reproduction and the not-for-profit research Foundation for Reproductive Medicine, both in New York, NY, USA.

Drs. Barad and Gleicher are co-inventors on several US patents hold patents related to androgen treatment in females (DHEA) with numbers US8067400B2, US8501718B2, and US9375436B2 (listed in USPTO/public records and assigned to American Infertility of New York). They also receive royalties from Nutraceuticals LLC, which has helped commercialize DHEA as a nutritional supplement. Dr. Gleicher is also a shareholder in Fertility Nutraceuticals and the owner of the Center for Human Reproduction. Dr. Albertini receives a stipend as Editor-in- Chief of the *Journal of Assisted Reproduction and Genetics* and receives consulting fees and travel support from Ferring Pharmaceuticals. Drs. Darmon, Gayete-Lafuente, Nicholas, Guijarro-Baude, and Patrizio report no conflicts of interest.

**TRIAL REGISTRATION NUMBER:**

N/A.

WHAT DOES THIS MEAN FOR PATIENTS? Preimplantation genetic testing for aneuploidy (PGT-A) is a lab test used during *in vitro* fertilization (IVF) to check whether an embryo has the usual number of chromosomes before it is transferred to the uterus. PGT-A is commonly used during IVF to help select embryos for transfer. It is often thought to be especially helpful for older women, who are more likely to produce embryos with chromosomal abnormalities. But whether a patient goes on to have PGT-A might also depend on ovarian reserve, which means how many eggs the ovaries are likely to produce. One common blood test for ovarian reserve is anti-Müllerian hormone (AMH). Higher AMH levels usually mean a better chance of producing more eggs and therefore embryos in an IVF cycle.

In this study, we looked at a large US IVF database and found that patients who did undergo PGT-A generally had higher AMH levels, and thus could produce more eggs and have more embryos available, than patients who did not undergo PGT-A. This difference was seen in all age groups and was especially noticeable in older women. This means that, in everyday IVF practice, patients who have PGT-A are possibly less fertile than those who do not. This is important when doctors and patients read studies about PGT-A, because some reported results may simply reflect the characteristics of the patients being studied, and not necessarily the effect of the test itself.

## Introduction

Preimplantation genetic testing for aneuploidy (PGT-A) is widely used in contemporary IVF, particularly among patients of advanced reproductive age, because increasing maternal age is associated with a higher frequency of embryonic aneuploidy and pregnancy loss (Hassold *et al.*, 1980; Simon *et al.*, 2018). In principle, PGT-A is intended to assist embryo selection by identifying embryos most likely to result in implantation and live birth per transfer and least likely to result in miscarriage (Practice Committee of the American Society for Reproductive Medicine and the Society for Assisted Reproductive Technology, 2024). For some patients and clinicians, this potential to guide transfer decisions and possibly shorten time to an ongoing pregnancy represents a meaningful clinical objective, even though the effect of PGT-A on broader treatment outcomes remains debated.

At the same time, the overall clinical value of PGT-A remains controversial. Randomized trials and observational studies have not demonstrated consistent improvement in cumulative live birth outcomes across unselected IVF populations (Munné *et al.*, 2019; Yan *et al.*, 2021), and the reported benefits have varied according to patient age, prognosis, study design, and outcome definition (Kucherov *et al.*, 2023; Harris *et al.*, 2025; Patrizio *et al.*, 2025). This distinction is important because PGT-A is fundamentally a selection tool: it may influence which embryo is transferred first, but it does not improve the intrinsic biologic competence of the embryos available within a cohort. Accordingly, the interpretation of both favourable and unfavorable outcome studies requires careful attention to the characteristics of the patients who do and do not undergo testing.

One patient characteristic that may be particularly important in this context is ovarian reserve. Anti-Müllerian hormone (AMH), a quantitative marker of functional ovarian reserve, is strongly associated with oocyte yield and embryo availability, and therefore with the practical feasibility of pursuing blastocyst biopsy and PGT-A. Patients with higher ovarian reserve may be more likely to proceed with PGT-A simply because they are more likely to generate enough embryos for selection, whereas patients with lower reserve may be less likely to do so, regardless of age-related aneuploidy risk. The objective of this study was therefore to determine whether ovarian reserve, measured primarily by AMH, is associated with utilization of PGT-A across age groups in a large national IVF registry, and to consider the implications of such utilization patterns for the interpretation of observational PGT-A studies.

## Materials and methods

### Study design and data source

This retrospective cross-sectional study used data from the SART Clinic Outcome Reporting System (SART CORS). Data were collected through voluntary submission, verified by SART, and reported to the Centers for Disease Control and Prevention (CDC) in compliance with the Fertility Clinic Success Rate and Certification Act of 1992 (Public Law 102-493). SART maintains HIPAA-compliant business associate agreements with reporting clinics. In 2004, following a contract change with the CDC, SART gained access to the SART CORS data system for the purpose of conducting research. Over 90% of all ART cycles in the USA are performed at SART-member clinics.

SART annually selects up to 10 clinics (approximately 2.5% of SART clinics) for an on-site validation visit using metrics and a blinded selection process to identify outlier clinics. Medical records are reviewed during the validation visit to verify the designation, outcome, and reporting of cycles. Clinics with significant systematic reporting errors undergo data correction. Six primary metrics and 26 secondary metrics are used for clinic selection. The metrics include low prospective reporting for both oocyte retrieval cycles and total cycles, high live birth rates in the various age groups, low cancellation rate, high percentage of total fertility preservation cycles, high percentage of embryo banking and oocyte banking cycles, high percentage of fertility preservation cycles where oocytes are thawed or embryos are transferred within a year, high percentage of deleted cycles, high percentage of cycles converted from IUI, and low percentage of cycles in which no embryos were suitable for transfer with and without preimplantation genetic testing (PGT). SART does not validate the accuracy of data entry fields, such as gonadotropin dosage, number of oocytes retrieved, number of fertilized oocytes, number of embryos cryopreserved, and PGT-A results, nor demographic fields, such as age and diagnosis.

Access to SART CORS data was approved by the SART Research Committee. The study was conducted using de-identified HIPAA-compliant data and was approved by the centre’s Institutional Review Board.

### Study population

We analyzed the first reported autologous ovarian stimulation cycle for each patient aged 21–46 years, performed between 1 January 2014 and 31 December 2021, from the most recent data available at the time of data request. Cycles involving donor oocytes, donor embryos, or gestational carriers were excluded. Cycles with missing key data or cycles using <150 IU/day of gonadotropins were excluded to reduce inclusion of natural-cycle or minimally stimulated IVF, which differ from conventional stimulation cycles in treatment intent, expected oocyte yield, and likelihood of proceeding to blastocyst culture or PGT-A. Analyses were stratified by age group and calendar year. Clinical pregnancy and live birth outcomes were not evaluated.

### Definition of PGT-A and control cycles

Cycles were classified as PGT-A cycles if preimplantation genetic testing for aneuploidy was recorded as performed on “all or some embryos”. Cycles recorded as not undergoing PGT-A served as controls. Cycles with unknown PGT-A status were excluded. Control cycles were not restricted by transfer type or embryo developmental stage. Fresh Day-3 transfers and cycles without available blastocysts were retained because excluding these cycles could preferentially remove patients with lower ovarian reserve or lower embryo yield; however, we acknowledge that registry data do not allow determination of original treatment intent in all such cycles.

### Ovarian reserve measures

The primary marker of functional ovarian reserve was the most recent AMH value measured within 1 year of the stimulation cycle. Additional measures included a clinical diagnosis of diminished ovarian reserve (DOR) as the reported indication for IVF, maximum baseline FSH, total gonadotropin dose, number of oocytes retrieved, ovarian sensitivity index (OSI; calculated as the number of oocytes retrieved per total gonadotropin dose), and the number of embryos suitable for cryopreservation or potential transfer. Associations among these parameters and their relationship to a clinical diagnosis of DOR were examined.

### Statistical analysis

We conducted a retrospective analysis of PGT-A utilization across AMH levels and age categories (<35, 35–37, 38–40, and ≥41 years). Multivariable logistic regression models were used to evaluate the association between AMH and the likelihood of undergoing PGT-A, adjusting for age, calendar year, race, total gonadotropin dose, gravidity, and prior ART. AMH was analyzed both as a continuous variable and dichotomized at 1 ng/ml for clinical interpretability. This threshold was selected *a priori* because it is widely used in clinical practice to distinguish reduced from more preserved ovarian reserve and has been associated with meaningful differences in oocyte yield and ovarian response (Rebar and Goje, 2018). Because dichotomization may oversimplify a continuous association, AMH was also modelled continuously. Prespecified interaction terms between age and AMH were tested. Subgroup analyses further examined how AMH influenced PGT-A use within each age group.

Continuous variables are reported as median (interquartile range [IQR], 25th–75th percentile) and compared using the Mann–Whitney *U* test. Categorical variables were compared using the *χ*^2^ test. A two-sided *P-*value < 0.05 was considered statistically significant. All analyses were performed using SAS software, version 9.4 (SAS Institute Inc., Cary, NC, USA).

## Results

Between 1 January 2014 and 31 December 2021, 636 012 autologous ovarian stimulation cycles were reported in the SART-CORS database. After applying the eligibility criteria, restricting age to 21–46 years and excluding gestational carriers, cycles with unknown PGT-A status, missing AMH, or total gonadotropin dosage <150 IU/day, we identified a final study group of 258 532 first autologous IVF cycles to be included in the analysis. Of these, 190 039 (74%) did not involve PGT-A and 68 493 (26%) did. The demographics of the population studied are reported in Table 1.

**Table 1.** Baseline characteristics of first reported autologous IVF cycles according to use of PGT-A.

Characteristic	PGT-A	No PGT-A	*P*-value
	*N* = 68 493	*N* = 190 039	
**Age, years**	36 [33–39]	35 [31–38]	<0.0001
**Age groups**			<0.0001
<35	25 121 (36.7%)	93 435 (49.2%)	
35–37	17 912 (26.2%)	41 509 (21.8%)	
38–40	16 458 (24.0%)	31 903 (16.8%)	
>40	9002 (13.1%)	23 192 (12.2%)	
**AMH, ng/ml**	2.2 [1.2–3.9]	2.0 [1.0–3.7]	<0.0001
**AMH groups**			<0.0001
<1 ng/ml	11 682 (17.1%)	47 317 (24.9%)	
≥1 ng/ml	56 811 (82.9%)	142 722 (75.1%)	
**Maximum FSH, mIU/ml**	7 [6–8]	7 [6–8]	0.1079
**Total FSH dose, IU**	3500 [2700–4500]	3300 [2475–4500]	<0.0001
**Oocytes retrieved**			<0.0001
0–4	5251 (7.7%)	29 890 (15.7%)	
5–8	12 963 (18.9%)	42 558 (22.4%)	
9–12	14 226 (20.8%)	38 722 (20.4%)	
>12	36 053 (52.6%)	78 869 (41.5%)	
**Ovarian sensitivity index (OSI)**	3.7 [2.0–6.5]	3.2 [1.5–6.1]	<0.0001
**Embryos transferred**	1 [1–2]	2 [1–2]	<0.0001
**Embryos cryopreserved**	4 [2–6]	1 [0–4]	<0.0001
**Usable embryos**	4 [2–6]	3 [1–5]	<0.0001
**Gravidity**			<0.0001
No	33 912 (49.5%)	103 267 (54.3%)	
Yes	34 581 (50.5%)	86 772 (45.7%)	
**Prior fresh ART cycles**			<0.0001
No	61 330 (89.5%)	165 809 (87.2%)	
Yes	7163 (10.5%)	24 230 (12.8%)	
**Prior frozen ART cycles**			0.0006
No	65 685 (95.9%)	182 806 (96.2%)	
Yes	2808 (4.1%)	7233 (3.8%)	
**Reason for ART: DOR**			<0.0001
No	51 690 (75.5%)	138 680 (73.0%)	
Yes	16 803 (24.5%)	51 359 (27.0%)	
**Ethnicity**			<0.0001
White	29 024 (42.4%)	89 727 (47.2%)	
Black	2588 (3.8%)	11 844 (6.2%)	
Asian	10 696 (15.6%)	19 117 (10.1%)	
Hispanic	3253 (4.7%)	10 954 (5.8%)	
Native American	102 (0.1%)	310 (0.2%)	
Mixed race	868 (1.3%)	1881 (1.0%)	
Unknown	21 962 (32.1%)	56 206 (29.6%)	

Data are presented as n (%) for categorical variables and median [Q1–Q3] for continuous variables. Categorical variables were compared using the chi-square test and continuous variables using the Mann–Whitney *U* test.

AMH, anti-Müllerian hormone; FSH, follicle-stimulating hormone; OSI, ovarian sensitivity index; ART, assisted reproductive technology; DOR, diminished ovarian reserve; PGT-A, preimplantation genetic testing for aneuploidy; Q1–Q3, 25th–75th percentiles.

AMH reporting increased over time, from 50% of cycles in 2014 to 75% in 2021. Patients undergoing PGT-A differed from those not undergoing PGT-A in several clinically relevant characteristics. Median AMH level was significantly higher in PGT-A cycles than in non-PGT-A cycles (median 2.2 [IQR 1.2–3.9] ng/ml vs 2.0 [IQR 1.0–3.7] ng/ml, *P* < 0.0001). Patients undergoing PGT-A were also older (median age 36 vs. 35 years, *P* < 0.0001), more likely to have >12 oocytes retrieved (52.6% vs. 41.5%, *P* < 0.0001), and had a higher ovarian sensitivity index (OSI: 3.7 [2.0–6.5] vs. 3.2 [1.5–6.1], *P* < 0.0001). Women who had four or more oocytes retrieved were significantly more likely to undergo PGT-A compared to those with fewer than four oocytes, with an odds ratio (OR) of 2.25 (95% CI: 2.18–2.32).

PGT-A cycles were associated with a greater number of embryos available for cryopreservation or potential transfer (median 4 [2–6] vs. 3 [1–5], *P* < 0.0001) and more cryopreserved embryos (median 4 [2–6] vs. 1 [0–4], *P* < 0.0001). The number of embryos transferred per cycle was lower in PGT-A cycles (median 1 [1–2] vs. 2 [1–2], *P* < 0.0001).

**Figure 1.**
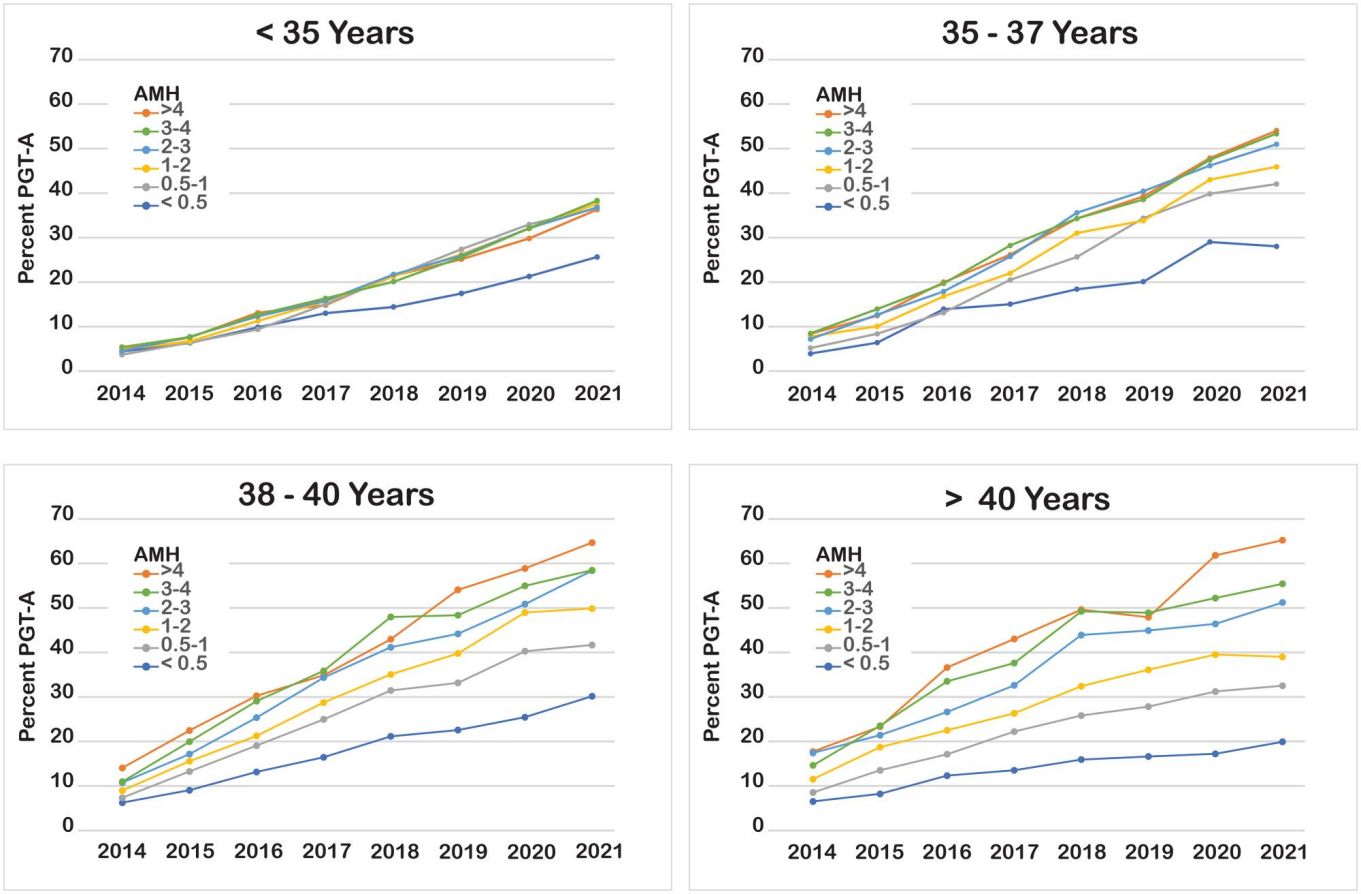
**PGT-A utilization by AMH category, age group, and reporting year in first autologous IVF cycles**. Percentage of first reported autologous IVF cycles in which preimplantation genetic testing for aneuploidy (PGT-A) was performed, shown by anti-Müllerian hormone (AMH) category and reporting year from 2014 to 2021. Separate panels are shown for patients aged <35 years, 35–37 years, 38–40 years, and >40 years. AMH categories are >4, 3–4, 2–3, 1–2, 0.5–1, and <0.5 ng/ml. All panels use the same *y*-axis scale (0–70%) to allow direct visual comparison across age groups.

PGT-A utilization increased with both age and AMH levels. Figure 1 illustrates this trend across six AMH categories within four age groups from 2014 to 2021. Across all ages, PGT-A use rose over the ensuing years, particularly among patients with higher AMH levels. The greatest divergence in PGT-A use by AMH level occurred in older patients, notably those aged 38–40 and >40 years, suggesting that ovarian reserve increasingly influenced clinical decisions about whether to pursue PGT-A in later reproductive years.

**Figure 2.**
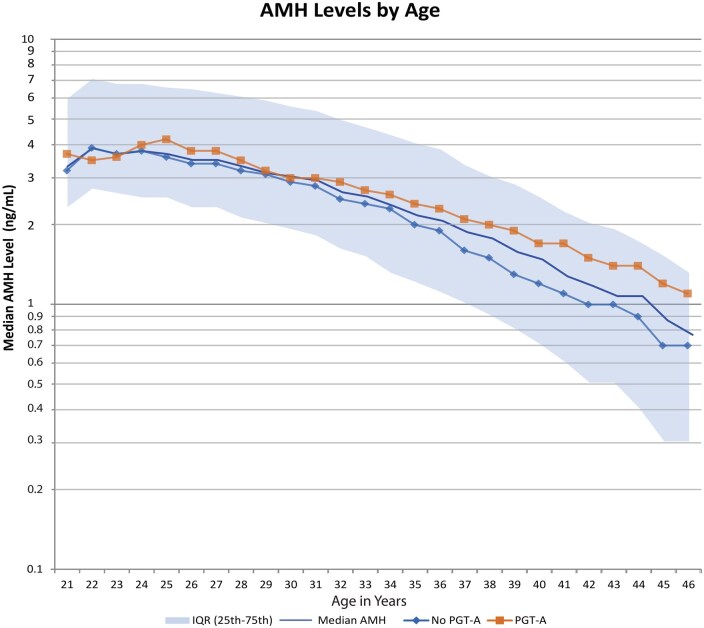
**Median AMH by age according to PGT-A status in first autologous IVF cycles**. Median anti-Müllerian hormone (AMH) levels by age among patients undergoing first reported autologous IVF cycles, stratified by whether preimplantation genetic testing for aneuploidy (PGT-A) was performed. Interquartile range (IQR: 25th–75th percentile) is displayed around the median. AMH declined with age in both groups but was consistently higher in patients undergoing PGT-A, with the difference becoming more pronounced at older ages. AMH is plotted on a logarithmic scale.

Figure 2 further highlights that AMH levels declined steadily with age but were consistently higher in patients who underwent PGT-A compared to those who did not. This difference in AMH widened significantly with advancing age, confirming that after age 35 years, patients with better ovarian reserve were more likely to undergo PGT-A.

In multivariable logistic regression models adjusted for age, calendar year, race, total gonadotropin dose, gravidity, and prior ART, lower AMH remained independently associated with a lower likelihood of undergoing PGT-A, when AMH was modeled as a continuous variable or when it was dichotomized at 1 ng/ml.

Across all age groups, patients with AMH ≥1 ng/ml were significantly more likely to undergo PGT-A than those with AMH <1 ng/ml, with adjusted odds ratios ranging from 1.41 to 1.50 (Table 2). The association persisted within each age stratum, supporting a consistent relationship between ovarian reserve and PGT-A utilization across reproductive age groups.

**Table 2.** Association between AMH category and likelihood of undergoing PGT-A, stratified by age group.

Age group	PGT-A, % (AMH <1 ng/ml)	PGT-A, % (AMH ≥1 ng/ml)	OR (AMH ≥1 vs. <1)	aOR (AMH ≥1 vs. <1)
<35	15.8%	22.0%	1.51 (1.44–1.58)	1.50 (1.42–1.58)
35–37	21.2%	32.8%	1.82 (1.73–1.90)	1.45 (1.37–1.53)
38–40	23.6%	39.0%	2.07 (1.98–2.16)	1.41 (1.34–1.48)
>40	18.6%	35.6%	2.42 (2.30–2.55)	1.41 (1.32–1.50)

Values for OR and aOR are shown as odds ratios with 95% confidence intervals. Adjusted odds ratios were adjusted for age, calendar year, race, total gonadotropin dose, gravidity, and prior ART.

AMH, anti-Müllerian hormone; PGT-A, preimplantation genetic testing for aneuploidy; OR, odds ratio; aOR, adjusted odds ratio; ART, assisted reproductive technology.

In a secondary analysis, AMH showed a more consistent association with recorded PGT-A utilization across age strata than the registry diagnosis of diminished ovarian reserve (DOR), supporting its use as the primary ovarian reserve marker in this study.

## Discussion

In this national registry analysis, we found a consistent association between utilization of PGT-A in autologous IVF cycles and ovarian reserve, as reflected by AMH levels and related stimulation characteristics. Across all age groups, patients who underwent PGT-A had higher AMH levels, greater oocyte yields, greater ovarian responsiveness to stimulation, and more embryos suitable for cryopreservation or transfer than those who did not undergo PGT-A. These differences became more pronounced with advancing age, indicating that use of PGT-A is more concentrated in a selected subgroup of patients with more favourable ovarian reserve characteristics. Because of the very large sample size, several between-group differences reached high statistical significance despite modest absolute magnitudes; accordingly, these findings should be interpreted primarily as evidence of consistent utilization patterns rather than large clinical effect sizes.

This distinction is clinically relevant because PGT-A is not applied uniformly across the IVF population. Rather, in routine practice, its use appears more common in patients with a higher likelihood of generating multiple embryos for selection. Our findings, therefore suggest that comparisons between PGT-A and non-PGT-A cycles in observational datasets should be interpreted in the context of differential patient selection, particularly with respect to ovarian reserve. For example, Rubio *et al.* (2017) excluded patients with fewer than four metaphase II oocytes as well as PGT-A cycles that failed to reach the blastocyst stage or yield euploid embryos. Within that selected population, cumulative live birth rates did not differ between groups, although time to pregnancy was shorter in the PGT-A cohort. However, exclusion of patients with poor ovarian response limits generalizability, and such findings should be interpreted with caution when considering the broader application of PGT-A based on age alone. In contrast, analyses that retained patients with lower ovarian reserve have reported longer times to live birth associated with PGT-A use (Mejia *et al.*, 2022).

Some patients may value PGT-A even though it has not been shown to improve cumulative live birth per initiated cycle. Patients who wish to avoid transfer of embryos considered less likely to result in live birth or more likely to result in miscarriage may regard PGT-A as a clinically useful tool for prioritizing embryos within a cohort. For such patients, potential advantages such as greater confidence in transfer selection and possibly a shorter time to an ongoing pregnancy may still be clinically relevant, even in the absence of demonstrated cumulative outcome benefit. Our findings, therefore, should not be interpreted as evidence that PGT-A lacks value in all clinical settings, but rather as indicating that patients who undergo PGT-A differ systematically from those who do not, especially with respect to ovarian reserve, and that this distinction should be considered in both patient counselling and interpretation of observational studies.

The age-stratified findings are also important. PGT-A utilization increased steadily between 2014 and 2021 across all age and AMH strata but remained consistently highest among patients with the greatest ovarian reserve. Notably, the widening AMH gap between PGT-A and non–PGT-A patients beginning around age 35 parallels the age range in which several recent observational studies have reported outcome differences associated with PGT-A (Munné *et al.*, 2019; Kucherov *et al.*, 2023; Harris *et al.*, 2025), raising the possibility that these findings may reflect differences in patient selection rather than treatment effect. As an embryo selection strategy, PGT-A ranks embryos within a cohort but does not modify intrinsic embryo quality or chromosomal constitution. In addition, embryo biopsy may result in embryo loss or misclassification, including exclusion of embryos with reproductive potential. Consistent with this, PGT-A has not been shown to improve cumulative live birth rates in younger women (Chang *et al.*, 2016; Kang *et al.*, 2016; Rubio *et al.*, 2017; Mejia *et al.*, 2022) and has, in some analyses, been associated with lower live birth rates (Chang *et al.*, 2016; Kang *et al.*, 2016; Mejia *et al.*, 2022; Kucherov *et al.*, 2023). Although higher live birth rates per embryo transfer have been reported among older women undergoing PGT-A (Chang *et al.*, 2016; Kang *et al.*, 2016; Rubio *et al.*, 2017; Mejia *et al.*, 2022), live birth rates per oocyte retrieval have not been shown to improve (Kang *et al.*, 2016; Munné *et al.*, 2019), consistent with randomized trials demonstrating no improvement in cumulative pregnancy outcomes when all viable embryos are ultimately transferred (Rubio *et al.*, 2017).

A recently published retrospective analysis reported increased cumulative pregnancy rates associated with PGT-A among older reproductive-age women (Harris *et al.*, 2025). However, the present findings suggest that differences in ovarian reserve between treatment groups offer a plausible alternative explanation, consistent with preferential use of PGT-A among patients with higher AMH levels.

Although our results do not evaluate treatment effectiveness and do not establish that any specific prior study is biased, they identify a utilization pattern that should be considered when interpreting observational comparisons and when designing future studies. This point is especially relevant in registry-based analyses, where treatment intent cannot always be determined. Some cycles classified as non-PGT-A may have involved an initial intention to pursue PGT-A but did not progress to blastocyst biopsy because of limited embryo development, whereas other cycles may never have been intended for testing. Conversely, some cycles recorded as involving PGT-A may have undergone testing only after embryo yield proved sufficient. These limitations are inherent to retrospective registry data and emphasize that our findings should be interpreted as patterns of recorded utilization rather than direct measures of physician intent or patient preference.

A secondary observation was that AMH appeared to relate more consistently to recorded PGT-A utilization than the registry diagnosis of diminished ovarian reserve (DOR). This is not unexpected, as diagnostic labels may be applied heterogeneously across clinics, whereas AMH provides a quantitative marker more directly linked to ovarian response and embryo yield. This finding should be interpreted as supportive of our use of AMH as the primary ovarian reserve measure, rather than as a primary endpoint of the study.

Our study has several strengths. It includes a large national sample of first autologous IVF cycles over multiple years, allowing robust assessment of PGT-A utilization across age groups in routine US practice. The analysis also focuses on objective ovarian reserve markers, primarily AMH, rather than relying only on diagnostic labels. At the same time, several limitations should be acknowledged. The cross-sectional design precludes causal inference. Residual confounding remains possible. Registry data do not capture all reasons for selecting or not selecting PGT-A, nor do they fully capture treatment intent. AMH reporting increased over time, and exclusion of cycles with missing AMH may have affected representativeness. Finally, embryo-level genetic outcomes, cumulative live birth, and time to pregnancy were not evaluated, and therefore, this study should not be interpreted as an assessment of PGT-A effectiveness.

Overall, these findings support the conclusion that ovarian reserve is closely associated with the use of PGT-A in clinical practice. This association does not by itself explain outcomes attributed to PGT-A, but it does define an important population context: patients who undergo PGT-A are, on average, not directly comparable to those who do not. Recognition of this distinction may improve both patient counseling and interpretation of future observational research.

## Conclusions

In this national registry analysis, utilization of PGT-A was closely associated with functional ovarian reserve, as measured primarily by AMH. Across age groups, patients undergoing PGT-A represented a selected subgroup with higher ovarian reserve and greater embryo yield than those not undergoing PGT-A.

These findings do not directly address treatment effectiveness, but they identify an important utilization pattern and potential source of selection bias that should be considered when interpreting observational studies of PGT-A. The issue appears especially relevant in older women, where patients undergoing PGT-A had higher AMH levels than age-matched counterparts who did not undergo testing. In light of randomized trials showing no improvement in cumulative live birth rates with PGT-A even in favourable-prognosis patients, assumptions that older women with limited embryo yields derive greater benefit in terms of live birth from PGT-A should be interpreted cautiously and require further study.

Future studies, including both observational analyses and randomized trials, should stratify or adjust for ovarian reserve using objective quantitative markers such as AMH to assess the impact of PGT-A more accurately.

## Data Availability

The data analyzed in this study were obtained from the Society for Assisted Reproductive Technology Clinic Outcome Reporting System (SART CORS) and are not publicly available. Access to this data is governed by SART and requires approval from the SART Research Committee, subject to applicable data use agreements and regulatory requirements. The authors do not have permission to share the data directly.
